# Prevalence of various Human Papillomavirus (HPV) genotypes among women who subjected to routine Pap smear test in Bushehr city (South west of Iran)2008-2009

**DOI:** 10.1186/1743-422X-7-65

**Published:** 2010-03-22

**Authors:** Keivan Zandi, Seyed Sajjad Eghbali, Rasool Hamkar, Shahnaz Ahmadi, Elissa ramedani, Iman Deilami, Heidar Aziz Nejad, Fatemeh Farshadpour, Zahra Rastian

**Affiliations:** 1 The Persian Gulf Tropical and Infectious Disease Research Center, Bushehr University of Mediccal Sciences, Bushehr, Iran; 2 Department of Virology, Faculty of Health Sciences, Tehran University of Medical Sciences, Tehran, Iran

## Abstract

**Background:**

Some genotypes of human papillomaviruses can infect the genital tract and they are important infectious agents which their oncogenicity is regardable. Thus the aim of this study was to determine the prevalence of various genital human papillomaviruses (HPV) among women being subjected to routine pap smear test in Bushehr city of Iran.

**Results:**

Based on the collected data, 11(5.5%) samples were detected positive for HPV DNA and 189(94.5%) samples out of 200 samples were detected negative for HPV DNA. Meanwhile 4(2%) samples detected positive for HPV DNA by PCR were detected positive for HPV by pap smear test as well. On the other hand 5 samples which were detected positive for HPV by pap smear test didn't have HPV DNA after being tested by PCR method. Among the 11 positive samples 7 samples were identified as HPV-16, 3 samples were HPV-18 and one was HPV-53.

**Conclusion:**

Regarding the prevalence of highly carcinogen genotypes of HPV in our study determination of genital HPV prevalence among the normal population of women of Bushehr city is recommended.

## Background

Worldwide, cervical cancer is the second most common cancer affecting women. Nowadays screening programs have reduced the mortality and morbidity of this disease, but 500,000 new cases of invasive cancer of the cervix are still diagnosed annually. Epidemiological and molecular studies have demonstrated that certain types of human Papillomaviruses (Highly carcinogen genotypes) are the major cause of most cases of cervical cancer [1].

Human papillomaviruses (HPVs) belong to Papillomaviridaefamily[2]. So far, 118 types have been identified according to their biological niche, oncogenic potential and Phylogenetic position. There are about 40 HPV viral types that are commonly found in the genital tract which are classified in the Alphapapillomavirus genus[3,4]. On the basis of molecular epidemiologic evidence, genital HPV types have been subdivided in to low-risk types and high-risk ones [5,6]. Several HPV types, such as HPVs 16, 18, 31, 33, 35, 39, 45, 51, 52, 56, 58, 59 and 66 have been implicated in cervical carcinogenesis (high risk types), whereas other types, such as HPVs 6 and 11, are frequently detected in benign lesions such as condylomata acuminate [1]. Women infected with high risk HPV types are considered to be at a higher risk for the development of cervical cancer than those who are not infected with HPV or are infected with low risk HPV types [6].

HPV-16 is the most prevalent type worldwide [7-9]. HPV18, 45, 31 and 33 are the next most prevalent types. In Asia, HPV58 and HPV52 are the next most common after HPV16 and 18[9]. HPV infection by multiple genotypes has been reported to occur in 10% to 20% of HPV-positive cases [10]. These epidemiologic findings in combination with follow-up studies often demonstrate the role of certain HPV types in cervical cancer development [1,11-13].

HPV DNA testing has been used as an adjunct to Pap smear cytology test in the diagnosis of cervical cancers to improve screening sensitivity and negative predictive value [11-13]. In fact, HPV DNA PCR is now recommended for patients with cytological abnormalities based on the new American College of Obstetricians and Gynecologists guidelines [14]. In addition, the importance of HPV genotyping in diagnostic practices is increasingly recognized in distinguishing high-risk from low-risk infections as well as in the development of cancer intervention strategies such as vaccine preparation[9,10]. Therefore, genotyping of HPV in clinical settings is regarded as an important diagnostic tool for cervical cancer, and also as means for providing valuable information necessary for its prevention and treatment.

In this study, we determined the prevalence of various HPV genotypes among the women subjected to routine Pap smear test in Abolfazl outpatient clinic of Bushehr University of Medical Sciences, Iran, by PCR and subsequent sequencing. Cytological diagnoses were also done on all samples based on the new Bethesda system.

## Methods

This study has been approved by the Ethical Committee of Bushehr University of Medical Sciences.

Two hundred samples were collected from women admitted at Abolfazl outpatient clinic in Bushehr and voluntarily subjected to gynecological examination by gynecologist from 2008 to 2009. Two Samples were collected from each patient by cytobrush: one for Pap-smear test for detection of cytopahic effects of HPV and another one for HPV DNA PCR assay which was stored at -20°C until PCR test time.

### DNA Extraction

All samples were subjected to centrifugation 2000 rpm for 5 minutes and precipitated cells were digested using digestion buffer containing proteinase K, followed by extensive extraction with phenol/chloroform [15]. The extracted DNA was stored at 4°C until tasted. DNA quality was evaluated by PCR using forward primer PCO3: 5'-ACACAACTGTGTTCACTAGC-3' and reverse primer PCO4: 5'-CAACTTCATCCACGTTCACC-3' that amplify a 110 bp product from the human β-Globin gene[12].

### HPV PCR

β-Globin positive samples were subjected to HPV PCR by forward primer GP5+: 5'-TTTGTTACTGTGGTAGATACTAC-3' and reverse primer GP6+:5'-AAAAATAAACTGTAAATCATATTC-3' for L1 open reading frame (ORF) that amplifies a 150 bp product from the HPV L1 ORF [12]. PCR was performed according to the procedure described previously[12]. Extracted DNA from HeLa cell line was used as HPV positive control and no DNA was added for negative control. Samples were subsequently subjected to agarose gel electrophoresis.

### DNA Sequencing Assay

HPV positive PCR products were subjected for automated sequencing (Kawsar Biotech Co., Tehran, Iran). Nucleotide sequences were aligned with CLUSTALW program using BioEdit software (BioEdit Sequence Alignment Editor Software, Department of Microbiology, North California State University) and confirmed by visual inspection. Genetic distance was estimated using the Kimura two-parameter matrix[16]. A phylogenetic tree was constructed by the neighbor-joining (NJ) method[17]. Bootstrap resampling and reconstruction were carried out 1000 times to confirm the reliability of the phylogenetic tree[18]. The analysis and calculated nucleotide differences within and between the isolate sequences were carried out by MEGA program, version 4[19]. The nucleotide sequences of HPV isolates reported in this article can be found in the GenBank database under accession numbers GU076513 through GU076523.

### Statistical Analysis

Data was processed by SPSS 16 Package program for statistical analysis (Chicago, IL, USA).

## Results

The result of HPV DNA PCR for collected samples revealed that there were 11(5.5%) samples with HPV DNA by using general primers for all genotypes of HPV. The result of genotyping based on MEGA software using which is based on genetic distances and phylogenetic tree informations (Table 1) showed that 7 samples out of 11 HPV DNA positive samples were detected as HPV-16, three samples were identified as HPV-18 and one sample was detected as HPV-53.

**Table 1:** Results of Sequencing for amplified L1 ORF of HPV Positive Samples

Results of Sequencing for amplified L1 ORF
TTTTGTTACTGTGGTAGATACTACACGGAGTACAAATATGTCATTATGTGCTGC CATATCTACTTCAGAACCTACATATAAAAATACTAACTTTAAAGAGTACCTACG ACATGGGGAGGAATATGATTTACAGTTTATTT

TGTTACTTTGGTAGATACTACACGCAGTACAAATATGTCATTATGTGCTGCCAT ATCTACTTCAGAACCTACATATAAAAATACTAACTTTAAAGAGTACCTACGACA TGGGGAGGAATATGATTTACAGTTTATTTTTCA

TTTTGTTACTGTGGTAGATACTACTCGCAGTACCAATTTAACAATATGTGCTTC TACACAGTCTCCTGTACCTGGGCGATATGATGCTACCAAATTTAAGCAGTATA GCAGACATGTTGAGGAATATGATTTACAGTTTATTTT

TTTTGTTACTGTGGTAGATACTACACGCAGTACAAATATGTTATTATGTGCTGC CATATCTACTTCAGAACCTACATATAAAAATACTAACTTTAAAGAGTACCTACG ACATGGGGAGGAATATGATTTACAGTTTATT

TTTTTGTTACTGTGGTAGATACTACACGCAGTACAAATATGTCATTATGTGCTG CCATATCTACTTCAGAACCTACATATAAAAATACTAACTTTAAAGAGTA

TTTTGTTACTGTGGTAGATACTACACGCAGTACAAATATGTCATTATGTGCTGC CATATCTACTTCGGAACCTACATATAAAAATACTAACTTTAAAGAGTACCTACG ACATGGGGAGGAATATGATTTACAGTTTATTTTTCA

TTTTGTTACTGTGGTAGATACTACACGCAGTACAAATATGTCATTATGTGCTGC CATATGTACTTCAGAACCTACATATAAAAATACTAACTTTAAAGAGTACCTACG ACATGGGGAGGAATATGATTTACAGTTTATTTTTCA

TTTGTTACTGTGGTAGATACTACTCGCAGTACCAATTTAACAATATGTGCTTCT ACACAGTTTCCTGTACCTGGGCAATATGATGCTACCAAATYTAAGCTGTATAG CAGACATGTTGAGGAATATGATTTACAGTTTATTT

TTTTGTTACTGTGGTAGATACTACACGCAGTACAAATATGTCATTATGTGCTGC CATATCTACTTCAGAACCTACATATAAAAATACTAACTTTAAAGAGTACCTACG ACATGGGGAGGAATATGATTTACAGTTTATTTTTCA

TTTGTTACTGTGGTAGATACTACTCGCAGTACCAATTTAACAATATGTGCTTCT ACACAGTCTCCTGTACCTGGGCAATATGATGCTACCAAATYTAAGCTGTATAG CAGACATGTTGAGGAATATGATTTACAGTTTATTT

TTTTGTTACTGTGGTAGATACTACACGCAGTACAAATATGTCATTATGTGCTGC CATATCTACTTCTGAACCTACATATAAAAATACTAACTTTAAAGAGTACCTACG ACATGGGGAGGAATATGATTTACAGTTTATTTTTCA

Results of pap smear showed that 9(4%) samples were detected as HPV positive meanwhile 4 of them were detected as HPV DNA positive by PCR method. On the other hand 7 samples which identified as HPV DNA positive by PCR method were negative for HPV infection when they investigated by pap smear test.

Mean of age for HPV infected women were 31.9 years old and it is concordant with the sexual active age which is very important for incidence of any kind of genital infections. The youngest one was 24 years old and the oldest one was 44 years old.

Based on clinical findings of HPV positive women we have found that five HPV infected women didn't have any clinical manifestations and there was no evidence for cervisitis and/or genital warts. Six women out of 11 HPV infected individuals have shown acute cervisitis but there was no sign related to genital warts and just one women showed the cervisitis and gentital warts together.

Besides, among five women who were detected positive for HPV infection by pap smear meanwhile they were detected negative for HPV infection by PCR method we have found that just two of them showed acute cervisitis without any genital warts manifestation.

By using SPSS software and regarding to the collected data, the sensitivity of pap smear test comparing to PCR method in our study was calculated 36.36%(95% Confidence Interval = 6.91-65.43). Besides the specificity value of our pap smear test was 97.35(95% Confidence Interval = 94.57-99.43).

## Discussion and Conclusion

Cervical cancer is an invasive cancer affecting approximately 500000 women each year of whom 80% live in developing countries. The vast majority of cervical cancer cases are caused by infection with certain genotypes of human papillomaviruses[1].

Pap smears for the detection of cytomorphologically abnormal cells have decreased the number of deaths from cervical cancer but have not eradicate the disease in any screened population to date[13,20]. cervical cytology is considered to be a very specific test for high grade pre cancerous leisions or cancer but even if the quality of collection and spreading of cells, fixation, and staining of smears, and reporting by well trained technicians and cytophatologists are good it's sensitivity is only moderate[21]. The result of meta analysis suggest that cytological screening has a very wide range of sensitivity to detect lesions[12]; for example, cytology is estimated to have mean sensitivity of 58% and a mean specificity of 69% in one study[12].

There is international consensus that "high risk" genotypes, including genotypes 16,18,31,33,35,39,45,51,52,56,58,59 and 66 can lead to cervical cancer. Infections with low risk genotypes, including 6, 11, ... can cause benign or low grade cervical tissue changes and genital warts[1,15,22]. HPV can not be cultured in vitro, thus analysis of DNA sequences can be used to identify HPV genotypes. PCR and *in situ *hybridization are two of the most sensitive methods[15]. The detection of HPV in cervical biopsies using these methods in conjunction with cytology could potentially improve screening for cervical cancer[15]. In this study we used the PCR method for initial detection of HPV and regarding to our facilities sequencing of the PCR products were performed by automated sequencing instead of in situ hybridization in order to genotyping of HPVs.

By using PCR, it was revealed that out of 200 samples, there were 11 (5.5%) positive samples for HPV DNA but there were only 4 (2%) positive samples with HPV related cytopathologic evaluation.

Based on results, seven samples (3.5%) were detected as negative for HPV by cytopathology method meanwhile they were positive for HPV DNA by PCR method. Such results express the false negativity of cytophatologic method. On the other hand five samples (2.5%) were recognized positive by cytopathology method for HPV, meanwhile they were negative for HPV DNA by using PCR method. This result confirmed the false positivity of cytopathologic methods. The sensitivity and specificity of cytopathology method in our study were 36.36% and 97.35% respectivly. Meanwhile in one study these criteria were reported as 58% and 64%, respectively [23]. In our study the prevalence of HPV was 11(5.5%) in 200 samples but in other study the HPV prevalence was 34.6 in Zagreb region and also the presence of HPV DNA was 99.7% in women with histologically confirmed SCC [24]. In one study in Mazandaran province (Iran) 33 (78%) cases were HPV positive based on PCR screening in cancerous group [15]. The difference in result of these studies could be due to type of patients and the sample size. As it is mentioned in results section five HPV infected women didn't show any clinical manifestations such as cervisitis or genital warts. Also, just in one HPV infected women genital warts was detected beside the acute cervisitis so we can conclude that HPV infections can occur without any significant symptoms and we couldn't rely on clinical manifestations and/or cytopathological examinations such as Pap smear test. Also, using colposcopy technique is recommended for further clinical investigations.

Out of 11 positive samples for HPV DNA 7 samples were HPV-16, determined by genotyping method, meanwhile 3 samples were HPV-18 and one sample was recognized as HPV-53. This showed that HPV-16 is the most prevalent type among high risk HPV in Bushehr city. Also the prevalent type of HPV in most studies was reported HPV-16 [7-9,21]. So as high-risk HPV types has correlation with cervical cancer, there is daily increase in need of screening test for women at risk of HPV infection [11-13,21]. Also, based on our and other studies results, using the PCR method for the detection of HPV infection is recommended.

## Competing interests

The authors declare that they have no competing interests.

## Authors' contributions

KZ and RH designed and conceived the PCR test. SSE performed the pap smear test ER, ID and SA have collected the samples. HA, ZR and FF co operated as lab staff for PCR performing. We confirm that all authors read and approved the final manuscript.
